# Resistance to anti-tubulin agents: From vinca alkaloids to epothilones

**DOI:** 10.20517/cdr.2019.06

**Published:** 2019-03-19

**Authors:** Werner Krause

**Affiliations:** Formerly Bayer AG, Project Management, Berlin 13353, Germany.

**Keywords:** Microtubules, tumor heterogeneity, mechanisms of resistance, cancer stem cells, biomarkers, pharmacokinetics, metabolism

## Abstract

This review describes the mechanism of action - inhibition of microtubules - and the most important mechanisms of resistance for vinca alkaloids, taxanes and epothilones. Resistance is a major problem in vinca and taxane chemotherapy and arises in most cases from overexpression of efflux pumps that transport the drugs out of the cancer cells and from modifications of the target, the microtubules, by overexpression of tubulin isotypes or by attachment of proteins to the ends of the microtubules so that the target is no longer recognized by the drugs. In some cases, however, this process can have the opposite effect, leading to sensitization, e.g., for vinca alkaloids in cases where taxanes are not or no longer effective. The link between resistance due to efflux pumps and the pharmacokinetics and metabolism of the drugs is also covered. Other types of resistance that are addressed include detoxification of drugs within the cancer cell and blockade of apoptosis, post-translational modifications of microtubules and other protein pathways, micro-RNAs, induction of oncogenes, and cancer stem cells, which, taken together, offer particularly multifold possibilities for preventing drug activity. The use of biomarkers for the prediction of clinical outcome and for the direction of future therapy is also addressed.

## Introduction

Anti-tubulin agents (ATAs) represent a class of anti-cancer drugs that are targeting one of the most fundamental processes in the organism, mitosis and cell division^[1]^. This had double-edged consequences. On the one hand it provided a boost of anti-cancer activity compared to previously available drugs, and on the other hand, it may elicit a host of defense mechanisms, both in the cancer cells leading to resistance and in normal cells resulting in side effects. Since the target is so essential to the existence and well-functioning of the organism, the number of mechanisms that can take over if one of them is blocked, is very high and until today only a limited number have already been extensively investigated. In this review, I will address the most important ones of them.

ATAs are directed against tubulins. Tubulins are the building blocks of microtubules, which are part of the cytoskeleton and which are essential for many physiological processes such as formation of the mitotic spindle and chromosome segregation during cell division, for migration or intracellular transport and for the construction of special structures such as cilia or flagella and their movements.

**Figure 1:**
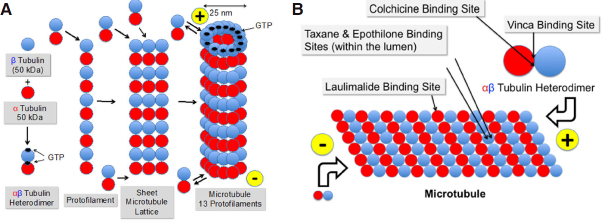
Formation of microtubules from α- and β-tubulin via αβ-heterodimers and protofilaments (A) and binding sites of colchicine, laulimalide, taxanes & epothilones, and vinca alkaloids (B)

Microtubules are tiny, very rigid, cylindrical, hollow tubes with a diameter of approximately 25 nm, composed of the two globular proteins, α- and β-tubulin with a total molecular weight of 50 kDa each [Figure 1]. In humans, eight isoforms of each α- and β-tubulin have been identified^[2]^. Some isotypes are specifically expressed in specialized cells and tissues. The predominant isotype is β-III in brain and testicular cells and β-I in other cell types. Isotype expression changes during development^[3,4]^. Post-translational modifications (PTMs) such as acetylation, phosphorylation, palmitoylation, sumoylation, *S*-nitrosylation, amination, glutamylation, glycylation, tyrosination, and detyrosination can lead to further diversification of microtubules^[5-9]^. Brain tubulin, which is a mixture of different tubulin isotypes with a majority of β-III, is heavily posttranslationally modified^[4,10-12]^. Most of the PTMs are reversible and their distribution is specific for the individual cell type^[5]^. The enzymes that introduce these modifications are essential to normal development^[13-15]^. Increased levels of tubulin modifications are a hallmark of cancer.

*In vitro*, and in the presence of GTP, one α- and one β-tubulin molecule are spontaneously and non-convalently linked together to form a heterodimer as soon as their concentrations exceed a critical value^[16]^. The *N*-terminal regions of α- and β-tubulin are able to bind GTP, which provides the energy needed for polymerization by hydrolysis to GDP and phosphate. Whereas the GTP binding site of β-tubulin is freely accessible and therefore can be used for energy purposes, the GTP binding site of α-tubulin is hidden in the interior of the heterodimer [Figure 1]. The heterodimers are available in the cells at relatively high concentrations of 1-10 μmol/L, compared to the total tubulin concentration, which is in the range of 20 μmol/L^[17,18]^.

Heterodimers are then lining up to form linear protofilaments and subsequently two-dimensional sheets. When 11-15 protofilaments have been linked together, they fold to form a tube, the microtubule^[19-21]^. The side with α-tubulin at the end is negatively charged, the other side, exposing β-tubulin, carries a positive charge. The negative end of the microtubule (α-tubulin) is attached to the microtubule-organizing center at the centrosomes and the positive end (β-tubulin) is growing into the cytoplasm of the cell by attaching new heterodimers^[22]^. The growth rate of microtubules varies between 5 and 20 μm/min^[22]^. However, there is a constant lengthening and shortening of the microtubules at both ends by adding and severing heterodimers^[23,24]^ called “dynamic instability”. The addition and elimination of heterodimers is slower at the minus end of the microtubule. There are periods where overall shortening of the microtubule is dominant, termed “catastrophe” and periods where lengthening is ongoing, called “rescue”. When the number of GTP-bound heterodimers at the microtubule end drops below a threshold level, the microtubule undergoes catastrophe and switches to rapid depolymerization^[21]^. This behavior is characteristic for non-equilibrium processes and constitutes the dynamic instability. The kinetics of these processes is controlled by a whole bunch of proteins^[1]^, termed microtubule-associated proteins (MAPs), such as for example γ-tubulin^[16]^. These proteins have a competing role for either stabilizing the microtubules, such as tau, MAP1, MAP2, MAP4, XMAP215 or destabilizing them, such as stathmin, XKCM1, XKIF2, and katanin^[25,26]^. They can address both sides of the microtubules and either promote or block microtubule polymerization or enhance or inhibit disassembly of the polymers. Reviews on this topic have been published by Janke *et al*.^[6]^, Farache *et al*.^[16]^, Ohi *et al*.^[27]^, Akhmanova *et al*.^[1]^, and Valiron *et al*.^[28]^. The dynamics of microtubules vary strongly during the cell cycle. Their turnover is slow in interphase cells. It can take minutes or hours depending on the cell type^[29,30]^. Mitosis accelerates this process up to 100-fold^[31]^. Anti-tubulin drugs with few exceptions decrease both the growing and shortening rate of the microtubules and the duration, increase the rescue frequency, and increase the time spent in a pause state^[32]^.

## Classes of anti-tubulin agents

Meanwhile, inhibition of tubulin is a well-established target in anticancer treatment^[33]^. Anti-tubulin agents come in two flavors, either stabilizing or destabilizing microtubules. Taxanes, and epothilones are microtubule-targeting agents that suppress microtubule dynamics by stabilizing microtubules and thereby interfering with the functioning of the mitotic spindle. As a consequence, the cell cycle comes to an arrest in rapidly proliferating cancer cells^[34]^, finally leading to apoptosis. Colchicine and vinca alkaloids destabilize the microtubules, leading to disassembly and cell death.

### Colchicine

The oldest drug that was investigated using the tubulin target is colchicine, which has been in use for more than 3000 years in the form of plant extracts. Colchicine has been approved by the FDA for the prophylaxis and treatment of gout flares and treatment of Mediterranean fever^[35]^. It is a natural product and was initially extracted from meadow saffron (*Colchicum autumnale*). Colchicine is able to block mitosis^[36]^. It tightly binds to the “colchicine binding site” [Figure 1] on heterodimers^[37]^, but not on microtubules. Following colchicine binding, the dimers are no longer able to form protofilaments or to attach to microtubules due to a change in conformation^[38]^. However, the required dose for cancer treatment was so high that the therapeutic index was too low for use in cancer indications.

### Vinca alkaloids

The discovery of the anticancer properties of vinca alkaloids in the late 1950s was a milestone in the development of cancer chemotherapy. Vinca alkaloids were originally obtained from the Madagascar periwinkle plant (*Catharanthus roseus*). They exhibit hypoglycemic and cytotoxic efficacy^[39]^ and have been used for treating diabetes and high blood pressure and as disinfectants. Five compounds have been approved as anticancer agents, vinblastine, vinorelbine, vincristine, vindesine, and vinflunine. Additionally, a liposomal formulation of vincristine, with sphingomyelin and cholesterol-based nanoparticles, has been approved to overcome the toxicity limitations of standard vincristine, the dose of which had to be capped at 2 mg for safety reasons.

The vinca binding site on microtubules^[40]^ is different from that of other anti-tubulin agents [Figure 1]. Vinca alkaloids induce tubulin to form alternate spiral polymers that will propagate into a microtubule GDP-containing lattice and dissociate microtubules by peeling spiral protofilaments^[32]^. These spirals are stable, equilibrium polymers that slowly dissociate. Vinca alkaloids are cell cycle phase-specific for the M-phase^[41]^. At high concentrations vinca alkaloids depolymerize microtubules and destroy mitotic spindles. The dividing cancer cells appear blocked in mitosis with condensed chromosomes. At low concentrations, they block mitosis more subtly, and cells die by apoptosis^[42]^. Similar to taxanes, vinca alkaloids exhibit antivascular and antiangiogenic effects, potently and rapidly inducing vascular disruption, thereby leading to tumor necrosis^[43]^. Vincristine, vinorelbine and vinblastine are on the WHO List of Essential Medicines.

### Taxanes

Paclitaxel (Taxol) is a microtubule-stabilizing diterpenoid, which had been isolated from the bark of Pacific yew (*Taxus brevifolia*) in 1971. At the beginning, only minimal amounts were available from the NIH since the cost of paclitaxel supply was prohibitive. The initial production of paclitaxel in the 1990’s required ten tons of bark from the Pacific Yew tree to produce one kilogram of paclitaxel^[44]^. For this purpose approximately 2000-4000 trees had to be cut down. A typical dose for breast cancer treatment needs 175 mg/m^2^ IV over 3 h every 3 weeks for 4 courses, which means a total of approximately 1.3 g per patient. One kg of paclitaxel therefore could - theoretically - treat approximately 700 patients. In practice, the number is much lower due to losses during manufacture, analytical testing, packaging, *etc*. Meanwhile the production costs could significantly be reduced by fermentation techniques.

*In vitro*, paclitaxel binds reversibly to the microtubule polymer in a 1:1 stoichometry relative to the heterodimer^[45,46]^ within the lumen of the microtubule, enhancing the polymerization of tubulin^[45,47]^. Initially, it was stated that the drug does not bind to the αβ-tubulin heterodimer^[48]^. Later, Field *et al*.^[49]^ could show that zampanolide and dactylolide also reacted with the same residue in unassembled tubulin, thus providing the first direct evidence of the existence of the luminal site in dimeric tubulin. Microtubules formed *in vitro* with paclitaxel present are extraordinarily stable. They even do not depolymerize upon addition of CaCl_2_, cooling down or diluting the solution^[50]^. Paclitaxel is able to induce polymerization in the absence of GTP, which normally is absolutely necessary^[51]^. Docetaxel is using the same binding site as paclitaxel, though with higher affinity^[52]^. Cell killing by paclitaxel is bi-phasically concentration dependent starting at 5-50 nmol/L where paclitaxel stabilizes the mitotic spindle and arrests the cell at mitosis. Blockade of cell proliferation then leads to apoptosis. At 1000 times higher concentrations of 5-50 μmol/L, polymerization of microtubules and bundle formation during interphase are increased, thereby inhibiting the initiation of S phase and finally leading to necrosis^[52,53]^. Paclitaxel additionally induces apoptosis by binding to the apoptosis-stopping protein B-cell leukemia-2 (Bcl-2) and thereby arresting its function. The cause for this effect is the similarity in the binding site of the natural Bcl-2 inducer, Nur77, on Bcl-2 and the taxane-binding site on microtubules^[54]^. Ganguly *et al*.^[55]^ proposed that paclitaxel rescues mutant cell division by inhibiting the detachment of microtubule minus-ends from centrosomes rather than by altering plus-end microtubule dynamics. Komlodi-Pasztor stated that the interference of paclitaxel with intracellular trafficking on microtubules is probably its most important function^[56]^. Additional effects of paclitaxel include the triggering of macrophages for TNFα and IL-1 production^[57]^ and its antiangiogenic activity by downregulating VEGF and Ang-1 expression in tumor cells and by increasing the secretion of TSP-1 in the tumor microenvironment^[58]^.

Taxanes with the approved drugs, paclitaxel (1991), docetaxel (1995), and cabazitaxel (2010) are a widely used class of anticancer agents. Galsky published a review for the latest approved drug, cabazitaxel^[59]^. Not approved but in clinical trials are tesetaxel and larotaxel^[60]^. Paclitaxel is also available as a protein-bound formulation, nanoparticle albumin-bound paclitaxel or nab-paclitaxel (Abraxane). Paclitaxel is on the WHO List of Essential Medicines. Approved cancer indications and dosing schedules are summarized in Table 1.

**Table 1:** Available anti-tubulin agents with their years of approval, main indications, dose ranges and types of therapy (monotherapy, combination partners)

Drug	Main indications	Dose	Combinations
Vinblastine 1961*	Hodgkin’s disease, non-Hodgkin lymphoma, histiocytic lymphoma, mycosis fungoides, testis, Kaposi’s sarcoma, choriocarcinoma, breast, kidney	3.7 mg/m^2^-18.5 mg/m^2^	Monotherapy, mechlorethamine, doxorubicin, vincristine, bleomycin, etoposide, dacarbazine, brentuximab, cisplatin, ifosfamide, methotrexate, mitomycine
Vincristine 1963*	Leukemias, lymphomas, myeloma, breast, lung, head & neck, sarcomas, Wilms’ tumor, neuroblastoma, retinoblastoma, medulloblastoma,	0.8 mg/m^2^-2 mg	Monotherapy, doxorubicin, carboplatin mechlorethamine, vinblastine, bleomycin, etoposide, cyclophosphamide, procarbazine, topotecan, dactinomycin, leucovorin, actinomycin D
Vindesine 1982***	ALL, CML, melanoma, breast	3 mg/m^2^-4 mg/m^2^	Monotherapy, cisplatin
Vinorelbine 1994*	NSCLC, Hodgkin’s disease, non-Hodgkin lymphoma, rhabdomyosarcoma, Wilm’s tumor, neuroblastoma	25 mg/m^2^-30 mg/m^2^	Monotherapy, cisplatin
Vinflunine 2009**	Urothelial carcinoma	280 mg/m^2^-320 mg/m^2^	Monotherapy
Vincristine Liposomal 2012*	Philadelphia chromosome-negative ALL	2.25 mg/m^2^	Monotherapy
Paclitaxel 1992*	Ovarian, breast, lung, gastric, Kaposi’s sarcoma	100 mg/m^2^-210 mg/m^2^	Monotherapy, cisplatin, doxorubicin
Docetaxel 1996*	Breast, lung, prostate, gastric, head & neck	75 mg/m^2^-100 mg/m^2^	Monotherapy, cyclophosphamide, cisplatin, 5-fluorouracil
Nab-Paclitaxel 2005*	Breast, lung, pancreas	100 mg/m^2^-260 mg/m^2^	Monotherapy, carboplatin, gemcitabine
Cabazitaxel 2010*	Prostate	20 mg/m^2^-25 mg/m^2^	Monotherapy
Ixabepilone 2007*	Breast	40 mg/m^2^	Capecitabine

Anti-tubulin agents first approved by FDA (*), EMA (**) or in other countries (***). ALL: acute lymphoblastic leukemia; CML: chronic myelogenous leukemia; NSCLC: non-small-cell lung carcinoma

### Epothilones

The latest drug class comprises the epothilones. The name was derived from their chemical structure as epoxide, thiazole and ketone. They initially raised high hopes due to their increased potency and by overcoming taxane and vinca resistance. Another advantage of the epothilones (excluding ixabepilone) over taxanes is their higher water solubility allowing formulations without additional solubilizers such as Cremophor EL, which might lead to hypersensitivity reactions. Epothilones were isolated from the myxobacterium *Sorangium cellulosum* in the 1990s^[61]^. According to their chemical structures, natural epothilones are characterized by the letters A-F. Only class B and D epothilones have been selected for development as anti-cancer drugs and as a basis for further chemical modification and optimization. Epothilone B is considerably more potent than epothilone A^[61]^. In general, the epothilones are more potent than paclitaxel by at least a factor of 10. The most potent epothilone is sagopilone with IC_50_ values in the sub-nanomolar range^[62]^. As with paclitaxel, cell killing by sagopilone is biphasic dose dependent. Low concentration treatment (2.5 nmol/L) resulted in aberrant cell division, aneuploidy and cell cycle arrest in G1 whereas high concentration treatment (40 nmol/L) led to mitotic arrest^[63]^. The gene expression profiles of low and high concentration phenotypes were substantially different from each other. Low concentration sagopilone led to p53 (TP53) transactivation in A549 lung cancer cells resulting in cell cycle arrest. Incubation with high concentrations of sagopilone was associated with upregulation of several genes involved in spindle assembly checkpoint and mitosis, indicating mitotic arrest. For both phenotypes, differences in the ability to undergo apoptosis were observed. Regarding resistance, sagopilone was tested in more than 100 different human tumor cell lines and a line resistant to the drug could not be found^[64]^. Epothilones and taxanes share the same binding site in the interior of the microtubule. Electron microscopy and molecular modeling suggest, however, that they do not share a common pharmacophore^[65]^. The interaction of epothilones with microtubules is functionally distinct from that of taxanes^[66]^.

Major compounds investigated in clinical trials were natural epothilone B (patupilone), the semisynthetic epothilone B derivative, ixabepilone (BMS-247550**)**, and the fully synthetic epothilone B derivative, sagopilone. In class D, natural epothilone D (KOS-862) and the epothilone D derivative, KOS-1584, were studied clinically. Ixabepilone is the only epothilone that was approved by the FDA in 2007 for the treatment of advanced breast cancer, either alone or in combination with capecitabine. The EMA declined approval in 2008 stating an insufficient benefit/risk ratio. Major side effects were limiting the use of the epothilones, particularly neurotoxicity (ixabepilone, sagopilone) or gastric toxicity (patupilone) and led to the discontinuation of development for patupilone and sagopilone. Ixabepilone is still on the market.

## Drug resistance

The use of vinca alkaloids and taxanes remains one of the major pillars in chemotherapy for a variety of tumor types. However, the majority of patients will develop progressive disease after initial response to therapy. Drug resistance therefore represents a major hurdle for the improvement of overall response and survival of cancer patients. In the following chapters resistance aspects will be covered for vinca alkaloids, taxanes and epothilones.

**Figure 2:**
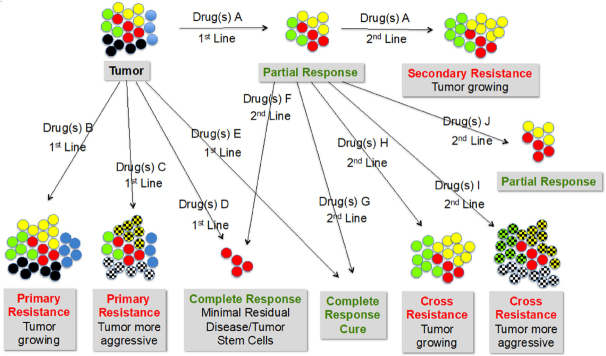
Heterogeneity of tumors and types of drug responses and resistance starting from no response at 1st line (primary resistance) via partial response to complete response with or without residual disease. In 2nd line treatment with a different drug the same type of reactions can be expected. In that case, no response means cross-resistance of the two different drugs is happening. Color codes: Red circles: cancer stem cells; other colors: different tumor cell types; cross-hatched circles: aggressive/metastasizing cell types

Drug resistance has a multitude of facets. Essentially it means that the tumor is not or not sufficiently or no longer responding to the therapy. The span of cancer treatment outcomes ranges from no response in 1st line drug exposure (primary resistance) via partial response to complete response with minimal residual disease to complete response without minimal residual disease (cure). In nearly all cases, retreatment is necessary, which could either mean repeating the first approach or moving to a different drug or combination of drugs. Even among taxane-naıve patients, primary resistance to taxanes is a critical factor for disease progression. More than one-third of patients with metastatic breast cancer do not respond to first-line anthracyclines or taxanes^[67]^. Cortes reported taxane resistance rates of up to 55% in anthracycline-pretreated patients and up to one-third in anthracycline-naıve patients^[68]^. Second-line, the same spectrum of outcomes can be expected. In case of no response with a different drug, cross-resistance is happening. Figure 2 summarizes some of the various possible scenarios.

### Tumor heterogeneity

The major assumption for the scenarios described in Figure 2 is the heterogeneity of tumors^[69]^. Each patient has his/her own tumor with different characteristics and therefore different therapy outcomes. The variabilities include but are not limited to different genetic, epigenetic, transcriptomic and proteomic properties. The genotypic changes include mutations, gene amplifications, deletions, chromosomal rearrangements, transpositions of the genetic elements, translocations and microRNA alterations^[70]^. The situation is even worse insofar as the same patient does not exhibit the same tumor over time and, even more important, the tumor at any time point x is a mixture of different cancer cells, even though clonally derived, with different characteristics and different sensitivity/resistance to treatment^[71]^. Genomic instability generates a great level of intercellular genetic heterogeneity in cancer.

### Types of resistance

Primary resistance can originate from a variety of possible mechanisms. It could either mean that the drug does not reach the target either at all or in sufficiently high concentrations in order to be able to elicit a cytotoxic effect. Or it does reach the target but it does not show any activity because the receptor is not recognized or not functioning properly.

According to Cree and Charlton^[72]^, resistance to anti-cancer drugs can be acquired by several mechanisms within neoplastic cells, defined as: (1) alteration of drug targets; (2) expression of drug pumps; (3) expression of detoxification mechanisms; (4) reduced susceptibility to apoptosis; (5) increased ability to repair DNA damage; and (6) altered proliferation. Not all of these mechanisms are important for ATAs. Since tumors are not homogeneous but a mixture of different clones, development of resistance is not a straightforward process but a universe of different activities at different levels. In many cases rapid resistance originates from multiple non-mutational, non-genetic mechanisms^[73]^.

### Pharmacokinetics and metabolism

The pharmacokinetics of the different drugs is an important factor in determining whether the substance is able to enter the cancer cell in sufficiently high concentrations and to approach the binding site. Table 2 provides a summary of the pharmacokinetics of vinca alkaloids, taxanes, and epothilones.

**Table 2:** Main pharmacokinetic parameters of anti-tubulin agents in humans

Drug	t_1/2_	Cl(tot)	Vd	BBB	P-gp	Metabolism	Ref.
Vinblastine	25 (23-85) h	0.74 L/h/kg (863 mL/min)	27.3 L/kg (2,047 L)	-	+	CYP3A4^1^	[74]
Vincristine	23-85 h	146 mL/min	215 L	-	+	CYP3A4/5^2^	[75]
Vindesine	20-24 h	NA	8.11 L/kg (608 L)	-	+	CYP3A4^2^	[76]
Vinorelbine	28-44 h	0.97-1.26 L/h/kg (1,280-1,680 mL/min)	25.4-40.1 L/kg (1,905-3,007 L)	+	+	CYP3A^3^	[77]
Vincristine Liposomal	7.7 ± 3.2 h	345 ± 177 (148-783) mL/h (6 ± 3 mL/min)	Vd = 3.6 ± 1.9 L Vdss = 2.9 ± 1.2 L	-	+	CYP3A4^2^	[41]
Vinflunine	40 h	40 L/h (667 mL/min)	2,422 ± 676 L	-	+	Esterases, CYP3A4^2^	[78]
Paclitaxel*	13-53 h	12.2-23.8 L/h/m^2^ (351-685 mL/min)	227-688 L/m^2^ (392-1,190 L)	-	+	CYP2C8, CYP3A4^2^	[79]
Docetaxel	11 h	21 L/h/m^2^ (605 mL/min)	113 L	-	+	CYP3A4/5	[79]
Nab-Paclitaxel	27 h	15 L/h/m^2^ (432 mL/min)	632 L/m^2^ (1,093 L)	-	+	CYP2C8, CYP3A4	[79]
Cabazitaxel	95 h	48.5 L/h (761 mL/min)	4,864 L	(+)	(-)	CYP2C8, CYP3A4, CYP3A5^4^	[79]
Patupilone	137 ± 70 h	14.0 ± 5.3 L/h (233 ± 88 mL/min)	224,2 ± 926 L	+	-	Esterases, CYP3A4/3A	[80]
Ixabepilone	35/52 h	616 mL/min	21-26 L/kg (1,575-1,950 L)	+	-	CYP2D6, CYP2C19, CYP3A4, CYP3A5^2^	[81]
Sagopilone	53 h	1,359 mL/min	4,607 L	+	-	Esterases, CYP3A4/3A, CYP2C19^2^	[62]
KOS-862	9.1 ± 2.2 h	9.3 ± 3.2 L/h/m^2^ (155 ± 53 mL/min/m^2^ = 268 ± 92 mL/min)	119 ± 41 L/m^2^ (206 ± 71 L)	+	-	CYP3A4, CYP2C9, CYP2C19, CYP2B6^2^	[82]
KOS-1584	21.9 ± 8.8 h	11 ± 6.2 L/h/m^2^ (183 ± 103 L/min/m^2^ = 317 ± 178 mL/min)	327 ± 161 L/m^2^ (566 ± 279 L)	+	-	CYP3A4^2^	[83]

*non-linear pharmacokinetics characterized by a disproportionately large increase in Cmax and AUC with increasing dose. Active metabolite(s): ^1^Desacetylvinblastine; ^2^not described; ^3^desacetylvinorelbine; ^4^N = 3, one of them docetaxel. t_1/2_: Terminal half-life; Vdss: volume of distribution at steady state; Vd: volume of distribution; Cl(tot): total plasma clearance; BBB: passage of the blood-brain barrier; P-gp: resistance due to P-gp overexpression. Assumptions for conversions: BMI = 1.73 m^2^, BM = 75 kg

All ATAs are extensively metabolized, mostly by P450 oxidizing enzyme systems, in particular CYP2C8 and CYP3A4. Any co-administered inducers of these enzyme systems, e.g., phenobarbital or carbamazepine, will therefore reduce the cancer drug concentrations in the blood significantly and thereby decrease or even eliminate the anti-tumor activity. This should, of course, be an avoidable case of “resistance” to the drug. On the other hand, any co-administered enzyme inhibitors, such as ketoconazole or fluoxetine will increase the drug concentrations and thereby could increase the efficacy, but at the same time increase toxicity.

The metabolism of vincristine, docetaxel, cabazitaxel, and ixabepilone by CYP3A5 is clinically relevant since this enzyme is polymorphically expressed. CYP3A5 is expressed in 10%-20% of Caucasians and approximately in 70% of African-Americans^[84]^. Differences in efficacy and tolerability therefore can be expected and may explain cases of “resistance” or drop-out due to side-effects. Likewise, CYP2C19, which metabolizes ixabepilone, sagopilone and KOS-862 exhibits genetic polymorphism. Approximately 3%-5% of Europeans and 15%-20% of Asians are poor metabolizers with no CYP2C19 function. Another example is CYP2C9, which is involved in the metabolism of KOS-862 and where allele frequencies for CYP2C9*2 range from 0% in Asians to 19% in Caucasians. The combined action of more than one of these polymorphically expressed enzymes as is the case with KOS-862 complicates the situation further.

ATAs in general do not require high peak concentrations of drug but rather long exposure of microtubules at a sufficiently high drug level^[41]^. Parameters for estimating this exposure are the terminal half-life of drug disposition in the blood, the total clearance and the volume of distribution [Table 2]. With 137 h, patupilone has the longest half-life and vincristine liposomal with 7.7 h has the shortest one. However, the latter number is misleading, since it had been determined after a long-lasting plateau of high drug concentration^[85]^. Therefore, the shortest half-life falls to KOS-862 with 9.1 h. The total clearance, where low values are preferred, is lowest for liposomal vincristine (6 mL/min) and largest for sagopilone (1,359 mL/min) and vinorelbine (1,280-1,680 mL/min). The largest volumes of distribution are exhibited by cabazitaxel (4,864 L) and sagopilone (4,607 L). Problems in this type of evaluation are the very high variability in PK parameters between patients, the non-linearity of PK in some cases, e.g., paclitaxel, and, the major point, that the decisive criterion is the concentration at the target receptor and not the concentration in blood. However, it might be assumed that a longer half-life will also lead to higher and longer-lasting accumulation of drug within the cancer cells. A further complicating factor is that some of the drugs, e.g., cabazitaxel, form active metabolites, which additionally contribute to activity. Cabazitaxel has three active metabolites, one of them being docetaxel. Paclitaxel has a volume of distribution of 392-1,190 L. It does not pass the blood-brain barrier. Cabazitaxel’s volume of distribution (4,864 L) is the highest of all ATAs. This drug is able to pass the blood-brain barrier as does sagopilone, with a Vd of 4,607 L. Probably, the high neurotoxicity of sagopilone can be explained by this effect. On the other hand, neurotoxicity is not an issue for cabazitaxel. In the case of cabazitaxel, the reason for not leading to neurotoxicity could be that the tubulin isotype distribution in brain is different from that in other tissues, with βIII-tubulin being predominant to which the taxanes do not bind whereas the epothilones do^[86-89]^. As can be seen from Table 2, passage of the blood-brain barrier goes parallel with being able to overcome P-glycoprotein (P-gp) resistance, which will be discussed in detail later in this review. The explanation is that the brain is protected by the same efflux pumps that are responsible for taxane drug resistance, mainly P-gp. Vinorelbine seems to be the only exception. It has been reported to pass the blood-brain barrier and to be subject to P-gp resistance.

ATAs are normally dosed in mg/m^2^ in order to compensate for differences in body surface, which certainly has advantages over a fixed dose. However, this dose regimen does not take into consideration the very high variability of pharmacokinetic parameters, which is independent of body surface or body weight and which might be much more important. Since all ATAs are extensively metabolized by the P450 system, the metabolic capacity of a patient is a factor that should be considered in a fully individualized dose regimen. Otherwise in patients with over-active enzymes, the bioavailable portion of the administered dose might be too low to be effective. In patients with less active or malfunctioning enzymes, the drug would be too toxic and would lead to discontinuation of therapy. Evaluation of the P450 metabolic status of a patient could lead to individual dose adjustments tailored to the needs of this patient.

### Efflux pumps

Taxanes and vinca alkaloids have been shown to be potent weapons in the fight against malignant tumors and are considered first-line therapy for the chemotherapy of many cancers. However, while patients often respond well to the first course of treatment, over time the response to drug exposure diminishes and the tumor may eventually become drug resistant. In some cases resistance can develop even across several classes of anti-cancer drugs, leading to cross or multidrug resistance^[90]^. The development of drug resistance limits the effectiveness of many anti-cancer agents and is an important contributor to cancer deaths.

In order to be effective, the cytotoxic drug has to be transported into the cancer cell and has to reach sufficiently high concentrations over a sufficiently long period of time. This can be achieved by using a concentration gradient between the exterior and the interior side of the cell membrane and by active transport. The concentration gradient alone is not sufficient for some types of anticancer drugs, such as nucleoside analogs, which need active transport into the tumor cell in order to achieve high intracellular drug concentrations^[69,91]^. There are three ATP-binding cassette transporter systems available, P-gp, multi-drug resistance-associated protein 1 (MRP1) and breast cancer resistance protein (BCRP/ABCG2)^[92]^. If the expression of these transporters is reduced, the uptake of the cytotoxic drugs into the cancer cell is diminished in those cases where they depend on active transport, such as nucleosides. If overexpression of the transporters occurs, drugs that had passively passed into the cancer cell by diffusion will be actively pumped out, if they are a substrate of the pumps. Both possibilities, over- and underexpression of active transporters, can lead to drug resistance, depending on the drug class., Since taxanes, vinca alkaloids and epothilones enter the cancer cells only via diffusion, underexpression of these transporters would constitute a beneficial situation. Overexpression of efflux pumps leads to resistance for taxanes and vinca alkaloids but not for epothilones.

A major obstacle in obtaining cytotoxic concentrations in cancer cells is the triggering of pumping systems that eliminate the drugs out of the cells thereby preventing the achievement of concentrations that are high enough to induce apoptosis. The most common form of clinical resistance is overexpression of the *MDR1* gene, which encodes the P-gp drug efflux pump. This membrane-associated ATP-binding cassette (ABC) transporter is overexpressed in many tumor cell lines, including tissues of the liver, kidney, and gastrointestinal tract. Overexpression of P-gp, encoded by the MDR1 gene, decreases intracellular drug levels, consequently limiting drug cytotoxicity. It is the most common mechanism involved in the development of resistance associated with poor response to microtubule-targeted agents including taxanes and vinca alkaloids and subsequent treatment failure^[93,94]^. Epothilones are not affected by P-gp induced resistance^[95,96]^.

Paclitaxel is a substrate for the P-gp drug efflux pump overexpressed in various multi-drug resistant cancer cells^[97,98]^. Vincristine and vinblastine are also affected by P-gp^[71]^. P-gp overexpression has been detected in up to 41% of multiple myeloma patient samples^[99]^. Inhibitors of P-gp, such as cyclosporin A, valspodar, and verapamil, have been tried to counteract overexpression of P-gp but have shown very limited activity in Phase III trials^[100]^. In an attempt to reverse multi-drug resistance, vincristine-loaded lipid nanoparticles conjugated to an anti-P-gp monoclonal antibody (MRK-16), showed greater cytotoxicity in resistant human myelogenous leukemia cell lines than control non-targeted particles. The response was attributed to the inhibition of the P-gp-mediated efflux of vincristine by MRK-16^[101]^. Cabazitaxel is a marginal substrate of P-gp^[102]^. It could be shown that P-gp, which is the main component of the blood-brain barrier, can be saturated by cabazitaxel so that passage of the barrier becomes possible. *In vitro*, cabazitaxel is not a substrate for the pumps, MRP1, MRP2, BCRP, OCT1 (organic cation transporter 1), OATP1B1 (organic anion transporting polypeptide 1B1) and OATP1B3.

Multidrug resistance protein 7 (MRP7; ABCC10) is an ATP-binding cassette transporter, which is able to transport amphipathic anions and confer resistance to docetaxel and, to a lesser extent, vincristine and paclitaxel^[103,104]^. An interesting approach is the use of the natural compound, β-elemene, which can reverse multidrug resistance mediated by the ABCB1 transporter^[105]^. The incubation of KB-C2 cells overexpressing the ABCB1 transporter with β-elemene (100 μmol/L) significantly augmented the antineoplastic efficacy of colchicine, vinblastine and paclitaxel when compared to KB-C2 cells incubated with these drugs alone. Glutathione-S-transferase (GSH) stimulates the ATP-dependent uptake and efflux of vincristine by MRP^[106]^. Cellular depletion of GSH decreases MRP1-mediated resistance to anticancer drugs.

In summary, all approved taxanes - with cabazitaxel being a special case due to possible saturation of P-gp - and all vinca alkaloids are substrates for the efflux pump, P-gp, leading to resistance, whereas the epothilones are able to overcome this resistance.

### Vault proteins

Once a drug has entered the cancer cell, the next obstacles could be vault proteins such as the lung resistance-related protein (LRP), a 150 kDa protein, which is overexpressed in multidrug resistant cells^[107]^. A 2-week treatment with sodium butyrate induced LRP and conferred resistance to doxorubicin, vincristine, etoposide, gramicidin D, and paclitaxel in SW-620 cells. Vaults are cytoplasmic organelles that were originally isolated in association with coated vesicles, but approximately 5% of the vaults are localized in nuclear pore complexes. Vaults are involved in the vesicular and nucleocytoplasmic transport of drugs. LRP is the major protein of vaults. Entrapment of the cytotoxic drug in vaults does not reduce the intracellular concentration of the drug but it makes it unavailable to target binding. Expression of LRP, but not P-gp or MRP, was an independent prognostic factor for predicting tumor response to standard chemotherapy and survival in patients with advanced ovarian cancer^[108]^.

### Detoxification mechanisms

Detoxification of cytotoxic drugs within the cancer cell can be accomplished by proteins of the glutathione S-transferase family (GST), which can be subdivided into three large groups, cytosolic, mitochondrial and microsomal proteins, known as MAPEG proteins^[69]^. Increased expression of GST leads to intracellular drug levels that are no longer cytotoxic.

### Target modifications

Another important line of defense of cancer cells is their ability to camouflage the target, in this case the microtubules. Altered tubulin isotype composition of microtubules has emerged as a feature of aggressive and treatment refractory cancers^[109]^. This could mean over- or underexpression of tubulin isotypes that are required as drug targets or the expression of isotypes that are not normally detectable in healthy tissue^[110]^. This behavior correlates with treatment response and patient outcome. Examples for aberrant overexpression include the βI-, βII-, βIII-, βIVa-, and βV-tubulin isotypes, which lead to aggressive clinical outcome and resistance to chemotherapy in many types of cancer. mRNA levels of βII-, βIII- and βV-tubulin isotypes were found to be significantly upregulated in paclitaxel and docetaxel resistant cells and significantly downregulated in vincristine resistant cells^[111]^. A modification in βIII-tubulin expression is the most extensively studied tubulin isotype mutation and it could be shown that subsequent resistance is a common feature for taxanes and vinca alkaloids.

The β-tubulin binding site of paclitaxel is located within the lumen of the microtubules. The drug needs to get there by diffusion, which is facilitated by the formation of hydrogen bonds with a β-tubulin serine moiety. The formation of this hydrogen bond is possible with all β-tubulin isotypes except βIII and βVI^[112]^. It was suggested^[2]^ that the presence of the unique residue, Ala218 in βIII-tubulin, may be a key reason why drug binding to βIII-tubulin is inhibited. Overexpression of this isotype therefore leads to resistance. Overexpression of βIII-tubulin has particularly often been described in taxane-resistant ovarian cancer^[86-88,113-118]^. But it is also seen in other tumor types such as lung, breast and gastric cancers^[119]^.

βIII-tubulin expression is controlled by estrogens in breast cancer cells but is influenced by exposure to hypoxia and poor-nutrient supply in ovarian cancer. Extensive microtubule mutations occur in hypoxic conditions^[109]^. Hypoxia-inducible factor 1-α (HIF1α) binds to an E-box motif located within the 30 UTR of TUBB3 and induces its expression in ovarian cancer cells^[120]^. Similarly, HIF1 and HIF2a interactions with overlapping HIF response elements within the TUBB3 3’UTR suppress and induce TUBB3 expression in glioblastoma in normoxia and hypoxia, respectively^[121]^. The expression of βIII-tubulin is also regulated by the interaction of the TUBB3 transcript with the RNA binding protein Hu antigen (HuR)^[122]^, which is involved in the recruitment of mRNA transcripts to polysomes and has been demonstrated to be involved in HIF1α stabilization. Oxidative stress depolymerizes microtubules and reduces the microtubule assembly rate^[123,124]^. βIII-Tubulin and the DNA damage repair enzyme excision repair cross-complementation group-1 co-operatively influence patient response to taxane combination therapy^[125]^.

In some but not all cancers, βIII-tubulin expression is purely a prognostic biomarker, predicting poor outcome of patients regardless of chemotherapy treatment. It enhances the incorporation of pro-survival kinases into the cytoskeleton and protects them from degradation. The associations of βIII-tubulin with survival kinase PIM-1, RNA-binding protein HuR, microRNAs are examples highlighting the functional complexity of this protein^[126]^. Ovarian clear cell adenocarcinoma and estrogen-receptor negative breast cancer, where βIII-tubulin overexpression indicates beneficial outcome of taxane-based chemotherapy, seem to be exceptions^[127,128]^. On the other hand, microtubules with overexpression of βIII-tubulin exhibit an increased sensitivity to epothilones, particularly to epothilone B^[129]^. The configuration of the pocket binding epothilone B (patupilone) in βIII-tubulin differs from that present in βI-tubulin, the most abundantly expressed β-tubulin^[130,131]^. Therefore, cells with high expression of βIII-tubulin appear more sensitive to patupilone^[129]^ explaining the increased effects of epothilones noticed in a large number of clinical trials conducted in patients relapsing after multiple lines of chemotherapy^[132]^. In other words, whereas overexpression of class βIII-tubulin plays a major role in taxane resistance, epothilones display their highest efficacy in βIII-tubulin overexpressing malignancies.

The expression of altered β-tubulin isotypes is triggered by certain oncogenes. Downregulation of βIII-tubulin by antisense oligonucleotides in paclitaxel-resistant A549-T24 cells resulted in a 40%-50% decrease in both βIII mRNA and protein levels, and was associated with a 39% increase in sensitivity to paclitaxel^[133]^. The expression of βIII-tubulin is regulated by TUBB3, a specific tubulin gene, which encodes the otherwise neuron-specific βIII-tubulin isotype. TUBB3 expression was increased in a resistant cell line [retinal pigment epithelial (RPE)-20] derived from untransformed RPE cells, but remained unchanged in four other cell lines after paclitaxel treatment^[33]^. TUBB3 levels alone therefore do not seem to be a good predictor of resistance.

Whereas increased βII-, βIII- and βIV-tubulin are associated with taxane resistance, decreased βIII-tubulin has been reported in vinca-resistant cell lines, together with increased microtubule stability^[134]^. βIII-Tubulin overexpression induces resistance to paclitaxel and vinorelbine, but does not affect the resistance to colchicine-site binding agents^[52]^.

Mutations in expression of β-tubulin isotypes are associated with altered sensitivity to ATAs. β-tubulin isotypes differentially affect the efficacy of ATAs in a cell type-dependent manner^[135]^. Overexpression of βII-tubulin and βIII-tubulin isotypes triggers resistance to taxanes in non-small cell lung cancer cells, whereas underexpression of βII-tubulin or βIVb-tubulin sensitizes NSCLC^[136,137]^ and pancreatic cancer cells to vinca alkaloids^[138]^. Underexpression of βII-tubulin also sensitizes NSCLC cells to epothilone B^[136,137]^. In ovarian cancer cells on the other hand, suppression of βII-tubulin does not sensitize the cells to taxanes or vinca alkaloids^[136]^. Conflicting results have been reported by Narvi^[139]^, who showed that βIII-tubulin but not βI-tubulin or βII-tubulin expression affected epothilone sensitivity in NSCLC and breast cancer cell lines. Likewise, overexpression of βIII- or βV-tubulin in CHO cells increases the resistance of these cells to paclitaxel^[140,141]^. Other studies indicate that overexpression of βI-, βII-, and βIVb-tubulin does not affect ATA resistance in these cells^[142]^. Overexpression of βIII-tubulin failed to induce resistance to ATAs in prostate cancer^[143]^. The epothilones do not seem to act in an identical manner but show individual differences. Whereas for ixabepilone βIII-tubulin overexpression reduces the efficacy^[144]^, for sagopilone resistant cell lines could not be found^[62]^.

βIII-Tubulin probably is a target of oncogenic pathways. Its expression is associated with ERG expression and TMPRSS2: ERG rearrangement in prostate cancer^[145]^. Jordan^[144]^ hypothesizes that altered β-tubulin isotype expression in cancer may protect cells from oxidative stress to trigger chemotherapy resistance and enable tumor progression by increasing the metastatic potential of cancer cells through migration and invasion. Parker *et al*.^[109]^ report that activation of the epithelial-to-mesenchymal transition (EMT) program is critical in regulating invasion and metastasis^[146]^ and that EMT re-programming is influenced by the tubulin isotype composition. βIII-Tubulin expression correlates with Snail expression levels and modulates the behavior of Snail overexpression during EMT in colon cancer cells^[147]^. Knockdown of βIII-tubulin expression with RNA interference agents caused modulation of colon cancer cell movement and a decrease in their ability to migrate and invade. Likewise, knockdown of ZEB1, an EMT transcription factor, in human breast cancer cells reduced β-tubulin isotype classes I, III, and IVB mRNA, whereas upregulation of ZEB1 was associated with increases in these isotype classes.

### PTMs

Another line of defense against cytotoxic drugs includes regulatory proteins, in particular alterations to tubulin through PTMs that affect regulatory protein binding, and altered expression or PTMs to tubulin-/microtubule-regulatory proteins^[51]^. The MAPs, tau, stathmin, MAP2 and MAP4 have been extensively investigated as triggers for cancer resistance^[148-150]^. They bind to and stabilize microtubules against depolymerization. High MAP expression is associated with resistance to taxanes^[148]^. Tau overexpression was associated with breast tumors that are resistant to paclitaxel^[150]^.

Stathmin and MAP4 affect the sensitivity of a cell towards paclitaxel. The overexpression or activation of stathmin and/or the downregulation or inactivation of MAP4 increases the microtubule dynamics and decreases their stability thereby resulting in resistance. On the other hand, the potency of microtubule destabilizers such as vinca alkaloids should be enhanced. Paclitaxel-resistant cell lines contain “hypostable” microtubules in which the equilibrium between the dimer and polymer is shifted towards the dimer^[151-153]^. These cells display increased resistance to polymer-binding drugs like paclitaxel, and increased sensitivity towards tubulin dimer-specific agents, such as vinblastine and colchicine. In paclitaxel-resistant cell lines, the equilibrium between weakly and highly dynamic microtubules is shifted towards the highly dynamic ones^[154-157]^. Two paclitaxel-resistant human ovarian cancer cell lines, 1A9PTX10 and 1A9PTX22, have been investigated. They were resistant to paclitaxel, but hypersensitive to vinblastine^[158]^. The paclitaxel-resistant cells also retained their sensitivity towards epothilone B.

### Micro-RNAs

Micro-RNAs (miRNAs) are another important factor in the establishment of resistance. miRNAs are short RNAs of 20-25 nucleotides that are involved in regulating gene expression, in particular protein-coding gene expression. They act via cleavage, destabilization or reduction of translation of mRNAs^[70]^. Exosomes function as vehicles delivering miRNAs from donating cells to accepting cells in the tumor microenvironment. A total of 22 miRNAs is concentrated in exosomes and correlates to resistance^[159]^. Dysfunction of miRNAs contributes to therapeutic resistance. For paclitaxel resistance, the following miRNAs are relevant, miR-17-5P for the target PTEN in ovarian cancer^[160]^, miR-30c for the targets VIM, IL-11 in breast cancer^[161]^, miR-125b for Sema4C and Bak1 in breast cancer, and miR-100 for mTOR, miR-145 for P-gp in ovarian cancer^[162]^, and miR-181a for PTEN in NSCLC^[163]^. For docetaxel resistance, miR-34a for the targets BCL-2 and CCND1 and miR-129-3p for CP100 have been identified. The mechanisms of miRNAs in drug resistance have been ascribed to the alteration of drug transporters leading to efflux of anticancer agents, modification of autophagy/apoptosis to enhance survival, promotion of growth factors to disturb associated signal pathways and activation of the EMT process and augmentation of cancer stem cell populations to promote metastasis^[161]^. A review on post-transcriptional gene silencing (RNAi) has been published by Naghizadeh^[164]^.

### Apoptosis inhibition

The last step in ATA cytotoxicity involves the death of the cancer cell, either by apoptosis, necrosis or autophagy. Apoptosis is controlled by ligands and cell death receptors such as FAS, TNF-R, caspase-3, -6, -7 and -8 and by linker proteins^[69]^. Within the mitochondria, Bcl-2 and AKT contribute as anti-apoptotic proteins and Bax, Bak and caspase-9 act as pro-apoptotic proteins. Overexpression of Bcl-2 and AKT and underexpression of Bax and Bcl-xl in cancer cells leads to resistance^[165]^. Likewise, p53 gene mutations can lead to apoptosis blockade^[166]^.

However, according to Murray *et al*.^[167]^, little consensus has been generated for reported associations between taxane sensitivity and mutated p53, or taxane resistance and overexpression of Bcl-2, Bcl-xL or NFκB. *In vitro* data support an association of spindle assembly checkpoint (SAC) defects with resistance. Clinical data have been limited and inconsistent. The most prominent finding is that pharmaceutical down-regulation of HER-2 appears to reverse the taxane resistance. This statement was opposed by Wang^[53]^ who published that proteins related to SACs such as survivin and MDA^[148,168,169]^, cell cycle-related proteins such as p53^[144,170]^, membrane receptors such as ErbB2^[148,171]^, and proteins related to apoptosis such as Bcl-2^[148,172,173]^ are all involved in taxane resistance.

### Oncoproteins

Sorcin is one of the most highly expressed calcium-binding proteins. It is an oncoprotein, which is co-amplified with the efflux pump, ABCB1, in multidrug-resistant cells^[174]^. Overexpression of Sorcin is found in many tumors resistant to taxanes and vinca alkaloids and inversely correlates with response and outcome of chemotherapy^[175]^. By transporting calcium into endoplasmic reticulum (ER) and mitochondria, Sorcin prevents ER stress and increases cell escape from apoptosis^[176-178]^. In MD-resistant tumor cells overexpressing Sorcin, the equilibrium between cell life and death is shifted towards proliferation. Silencing of Sorcin reverses MDR in many tumor cell lines^[175,179-181]^. Another interesting aspect of Sorcin is its ability to directly bind drugs like vincristine or paclitaxel and thereby decreasing their effective concentrations in the cancer cell.

Another example for this type of proteins is S phase kinase associated protein 2 (SKP2), which is an oncoprotein and a multicomponent RING-type E3 ligase that degrades many tumor suppressor proteins, such as p27, p16, p21, p57, E2F-1, TOB1, RBL2, cyclin D/E, BRCA2, FOXO1 and RASSF1A^[182]^. SKP2 is closely linked to paclitaxel sensitivity. High expression levels of SKP2 are a biomarker for poor cancer prognosis.

### Other protein systems

The ubiquitin-proteasome system is essential for protein degradation in many processes, including cancer progression and chemoresistance^[183]^. F-box proteins, which are part of this system, have the ability to control the degradation of several crucial protein targets associated with drug resistance, the dysregulation of which may lead to induction of chemoresistance in cancer cells^[182]^. Out of the three F-box categories, FBXW, FBXL and FBXO, FBXW7 is a well-established FBXW subfamily protein, which plays a key role as tumor suppressor in the occurrence of many forms of cancers. Loss of FBXW7 leads to increased resistance of colon cancer cells to paclitaxel^[184]^. FBXW7 primarily exerts its antitumor functions by regulating the degradation of an entire network of proteins, including cyclin, c-Myc, c-Jun, Notch, presenilin, and myeloid cell leukemia-1 (Mcl-1), many of which have oncogenic functions^[182]^. For example, the inhibition of Mcl-1 restores sensitivity towards paclitaxel and vincristine-induced cell death. Therefore, the downregulation of FBXW7 protein levels may contribute to tumor progression and chemoresistance. Other targets of FBXW7 include multidrug resistance-associated protein, cryptochrome 2 and inhibitor of growth 5. The tumor suppressor gene p53 directly targets FBXW7 and promotes the transcription of FBXW7 mRNA^[185]^. FBXW7 is also affected by dysregulation of the miRNA pathway, in particular by miR-25 and miR-223^[186,187]^. Overexpression of miR-223 decreases FBXW7 expression and the sensitivity to chemotherapy.

Mozzetti *et al*.^[188]^ reported that the Gli family of transcription factors, which are effectors of Hedgehog signaling, are overexpressed in patupilone resistant cells and that treatment with a specific Gli1 inhibitor (GANT58) made cells more susceptible to treatment, partially reversing drug resistance. Gli1 overexpression also led to an increase in hepatocyte growth factor, which is a stimulator of mitogenesis and cell motility and plays a role in angiogenesis and cancerogenicity.

β-Transducin repeats-containing protein (β-TrCP, FBXW1) degrades nuclear factor of kappa light polypeptide gene enhancer in B-cells inhibitor, alpha (IκBα) and β-catenin^[182]^. IκB is the inhibitor of nuclear factor kappa-light-chain-enhancer of activated B cells (NF-κB), a tumor suppressor and β-catenin is a downstream protein of the Wnt pathways. βTrCP acts as an oncoprotein in colorectal cancer and as a suppressor in gastric cancer. As a last example in this series, FBXO15 knockdown has been reported to cause P-gp accumulation leading to enhanced vincristine resistance^[189]^.

### Sphingolipids

The sphingolipid-mediated sphingosine-1-phosphate (S1P) pathway, including sphingolipid metabolites, regulates multiple cellular processes including proliferation, neovascularization, migration, invasion, and metastasis by controlling cell signal transduction networks that contribute to both tumorigenesis and tumor progression^[190]^. Ceramide, sphingosine, and S1P, which are rapidly interconverted in response to various stimuli, are the core players of this pathway, promoting growth, motility and angiogenesis (ceramide) or mediating anti-proliferative and cytotoxic stress responses including apoptosis, cell cycle arrest, lethal autophagy, and growth suppression (sphingosine, S1P). Chemotherapy or other stress factors increase the levels of ceramide and sphingosine. In normal cells under normal conditions, sphingosine and S1P levels are tenfold lower than the level of ceramide. S1P can be moved out of the cell by active transport and may affect metastasis by inhibiting or enhancing migration and invasion in a cell-type- and concentration-dependent manner. S1P signaling has been identified in the top three most transcriptionally altered pathways following chemotherapy of ovarian cancer^[191]^. Increased expressions of ceramide transport protein (CERT), SPHK1, SPHK2, and glucosylceramide synthase have been associated with resistance to paclitaxel and apoptotic response^[192]^. The level of ceramide is lower in ovarian tumor cells than in normal ovarian tissue and is further attenuated in paclitaxel-resistant compared to paclitaxel-sensitive ovarian cancer cells, skewing the ratio of ceramide: S1P in favor of the anti-apoptotic lipid S1P in tumor cells^[193]^. Therapeutic approaches should aim to promote ceramide accumulation (e.g., via ceramide analogs or mimetics or by co-administration of paclitaxel and ceramide in nanocarriers) to induce apoptosis and suppress S1P accumulation (e.g., by neutralizing antibodies), to inhibit tumor growth and overcome drug resistance.

### Cancer stem cells

The last point that I will address is cancer stem cells (CSCs). CSCs were first found in 2003 by Al-Hajj *et al*.^[194]^ and Singh *et al*.^[195]^ who identified them using the cell surface markers CD34+/CD38- in breast and brain cancer samples, based on work by Lapidot *et al*.^[196]^ on acute myeloid leukemia. CSCs are the prototypes of therapy resistant cells. They make up as few as 1% of the total tumor cells, making them difficult to detect and study^[197]^, but could as well reach 25%^[198]^. On the other hand, as few as 100 cancer stem cells were able to form tumors in non-obese diabetic/severe combined immunodeficient (NOD/SCID) mice^[194]^. The expression of CSC surface markers is tissue type-specific, even tumor subtype-specific^[199]^. CSCs are defined by their ability to generate more SCs (self-renewal) and to produce cells that differentiate^[200]^. Asymmetric cell division achieves both tasks, as one progeny retains SC identity and the other undergoes rounds of cell division and subsequent post-mitotic differentiation. Cancer cells gain stem-like characteristics through EMT. EMT is driven by transcription factors, including SNAI1/2, ZEB1/2, or TWIST1/2, which increase the invasiveness of epithelial cells. Further involved pathways include Wnt, Hedgehog, Hippo, LATS 1/2, and miRNA. CSCs reside in niches, which are specialized microenvironments located within each tissue. Since the ABCB1 and ABCG2 efflux transporter genes are expressed in both normal stem cells and most tumor stem cells^[201]^, any therapy that spares normal stem cells will also spare CSCs. Likewise, inhibition of drug transporters may also cause toxicity of the patient’s normal stem cells, particularly those of the bone marrow.

The expression of a major glycosaminoglycan in the extracellular matrix, hyaluronan (HA), and its receptor, CD44, a cell surface marker for both normal and cancer stem cells, are tightly linked to MDR and tumor progression^[202]^. In breast and ovarian cancer cell lines, HA-CD44 interaction may activate the stem cell marker, Nanog, which can further activate the expression of pluripotent stem cell regulators (Rex1 and Sox2) and Stat-3-mediated ABCB1 gene expression. In addition, HA-CD44 binding may form a complex with ankyrin, the downstream effector of CD44. This complex formation results in an efflux of chemotherapeutic drugs.

In Figure 2, CSCs are characterized as red circles. Most likely, “minimal residual disease” is nothing else than CSCs. CSCs exhibit the capabilities of self-renewal, differentiation and tumorigencity. There are several theories for their formation. One states that CSCs arise from normal stem cells following genetic or environmental alterations, another one proposes the reverse process that normal somatic cells become malignant and stem-cell like via genetic or environmental alterations. The situation very much reminds me of HIV treatment where standard therapy is able to keep HIV to a minimum, including non-detectable RNA blood levels, and as soon as treatment is stopped, dormant HIV cells start replicating and there is a burst of disease. This is the reason that until now, cure of HIV infection has not been achieved. Current HIV drugs only inhibit proliferation of the virus but do not kill infected cells. In a recent study, we were able to show that HIV-infected cells can be killed by an antibody directed against CD52, which is expressed on normal and on HIV-infected cells^[203]^. If we apply these findings to cancer, CSCs would be “dormant” cancer cells that survive any tumor treatment and when they awake lead to progression and metastases. If that should be the case, then there would be two ways of fighting CSCs, either the use of as yet unknown drugs that are able to address dormant cells, potentially via their specific cell surface markers and using a kill mechanism that applies to dormancy, or by forcing the cells out of their hibernation and thereby induce replication, which should make them sensitive to conventional ATAs.

### Summary and outlook

In summary, although the taxanes of the first generation, paclitaxel and docetaxel, were revolutionary new drugs and soon became blockbusters, they still had quite a number of drawbacks, such as: (1) extremely low water solubility, which required the use of solubilizing agents (polyoxyethylated castor oil or polysorbate) leading to hypersensitivity reactions with the need for premedication and even modification of drug pharmacokinetics; (2) poor metabolic stability resulting in highly variable pharmacokinetics and poor oral bioavailability with the consequence of long-lasting intravenous infusions and becoming substrates of P450 enzyme inducers or inhibitors; (3) serious dose-limiting toxicities such as myelosuppression, cumulative peripheral sensory neuropathy, and allergic reactions; and (4) most importantly, development of resistance due to a number of different mechanisms, the foremost being induction of efflux pumps and target modifications, mainly βIII-tubulin overexpression.

The development of novel formulations, such as nab-paclitaxel, somewhat relieved the issue of low aqueous solubility and surprisingly opened up new indications such as pancreatic cancer but did not change the resistance problems. The development of other formulations, e.g., nanocarriers, liposomes, cyclodextrin-containing systems, *etc*. is ongoing^[204,205]^ and might improve tolerability of the drugs but, again, most probably not resistance. Currently, the development of new nano-formulations is a very active area but drug combinations of taxanes with compounds of different mechanisms of action appear to be the most active field of research in the fight against resistance.

Cabazitaxel is a 2nd generation taxane with improved characteristics in terms of pharmacokinetics and metabolic stability and moving in the direction of eliminating the P-gp efflux pump resistance. More compounds have been in clinical trials^[206]^, such as tesetaxel, larotaxel, ortataxel, and milataxel, which are orally bioavailable, and - some of them - overcome P-gp efflux pumps, representing steps in the right direction. However, except for tesetaxel, the development of the other compounds has been discontinued due to toxicities. Preclinically, more taxane analogs are currently being investigated, but will not be covered in this review.

Whereas for other anticancer drug classes tumor-targeting drug conjugates have become the new hype, for the taxanes, there is only one compound under investigation, ANG-1005, which is a paclitaxel-Angiopep-2 conjugate^[207]^ aiming at brain tumors. ANG1005 crosses the blood-brain barrier and enters the cancer cells via receptor-mediated transcytosis of the low-density lipoprotein receptor-related protein 1 (LRP1), which is upregulated in some cancers. Within the cancer cell, esterases will then cleave the conjugate and release paclitaxel. If the intracellular drug concentrations are high enough, P-gp overexpression might no longer be relevant for this drug, which is currently preparing for Phase III.

Regarding vinca alkaloids, the situation is similar to the taxanes. A liposomal formulation of vincristine has been developed and approved in order to improve the shortcomings of the original drug, such as solubility issues and dose limiting toxicity. A new compound, vinflunine, was approved in 2009. Likewise, the synthesis of novel vinca compounds with the aim of maximizing potency and minimizing resistance is ongoing and looks quite successful.

The epothilones described above might have been abandoned too early. The toxicity problems observed in the clinical development that led to discontinuation may be attributable in part to studying worst-case scenarios in most of the trials, i.e., heavily pretreated patients that no longer responded to any other drugs and which were then moved to monotherapy with epothilones. There is also still insignificant knowledge why the dose-limiting toxicities of the epothilones were so strikingly different, for example diarrhea for patupilone and neurotoxicity for sagopilone.

Another aspect is that at the time of development of the epothilones, knowledge about biomarkers was still limited and could not be used for patient selection. Epothilones overcome both efflux pump-related resistance and tubulin isotype-related resistance, in particular overexpression of βIII-tubulin, which are the two major stumbling blocks for taxanes. If this knowledge would have been available and been applied to patient selection, maybe the epothilones would have taken another course and not been abandoned. Likewise, the combination of epothilones with other anticancer drugs with different mechanisms of action was not sufficiently taken into consideration. In particular, platinum-containing drugs might have been appropriate partners for combination.

Alternative ATA drug classes that are currently under preclinical or clinical investigation include natural compounds isolated from marine sponges or plants. Laulimalide (fijianolide) was originally obtained from the sponge *Cacospongia mycofijiensis*^[208]^. It binds at a microtubule site different from that of other ATAs located on the exterior of the microtubules^[209]^ [Figure 1]. Laulimalide is an inhibitor of cellular proliferation in cancer cell lines with IC_50_ values in the low nanomolar range. The drug causes cells to arrest in the prometaphase preventing formation of bipolar spindles and increasing kinetochore tension in preformed spindles^[210]^. Laulimalide is also active in multi-drug resistant cancer cell lines overexpressing P-gp or presenting mutated tubulin isotypes^[211]^ and is both effective in taxoid-reistant cell lines and synergistic with taxoids^[212]^.

Peloruside A is a macrocyclic compound isolated from the New Zealand marine sponge, *Mycale* *hentscheli*. It has antimitotic, microtubule-stabilizing and antineoplastic activity^[213]^. It appears to share the same binding site as laulimalide on the exterior of the microtubules. Peloruside A may be particularly useful for anti-angiogenesis therapy due to its high efficacy in blocking endothelial cell migration^[214]^.

Discodermolide (XAA296A) was isolated from the deep-sea sponge, *Discodermia dissoluta*^[215]^. The compound is one of the most potent natural promoters of tubulin assembly and competes with paclitaxel for binding, but with higher affinity. Due to its improved water solubility, the formulation problems encountered with paclitaxel can be avoided. Discodermolide is effective in paclitaxel- and in epothilone-resistant cell lines^[216]^ and was investigated in a Phase I clinical study showing good tolerance and non-linear pharmacokinetics^[217]^.

Zampanolide was obtained from the marine sponge, *Fasciospongia rimosa*^[218]^. The compound competes with paclitaxel for binding to microtubules^[219]^. However, in contrast to paclitaxel, zampanolide’s binding is covalent at residues N228 and H229. Alkylation of N228 and H229 was also detected in αβ-tubulin dimers^[220]^. Efforts to generate a zampanolide-resistant cell line were unsuccessful. Zampanolide could be shown to be an effective inhibitor of migration of human umbilical vein endothelial cells and fibroblast cells (D551) and inhibits cell growth in paclitaxel- and epothilone-resistant cells^[221]^.

Taccalonolides are natural, highly oxygenated pentacyclic steroids of tacca plants (Polynesian arrowroot). The first compound was isolated from *Tacca leontopetaloides*^[222]^. Meanwhile a large number of taccalonolides have been isolated from different tacca genus, designated A-Y. Bioassay-guided fractionation led to the identification of taccalonolides A and E as microtubule stabilizers. They retain efficacy in cells containing mutations in the paclitaxel binding site as well as those expressing P-gp, the βIII-isotype of tubulin or the MRP7 drug efflux transporter. Since the taccalonolides A and E were unable to interact directly with microtubules or to enhance the polymerization of purified tubulin and their *in vivo* efficacy was much higher than *in vitro*, it was proposed that they actually are prodrugs. In contrast, the significantly more potent natural taccalonolide AF and the semi-synthetic taccalonolide AJ directly interact with microtubules via covalent binding^[223]^. Taccalonolides are able to displace paclitaxel from its binding site indicating that they bind at or near the luminal and/or pore taxane binding site. Their activity is synergistic with paclitaxel and with laulimalide^[224,225]^.

PM050489 and its dechlorinated analog, PM060184, are natural polyketides and were isolated from the Madagascan sponge *Lithoplocamia* *lithistoides*^[226]^. They are highly potent microtubule inhibitors that impair mitosis by binding with nanomolar affinity to unassembled αβ-tubulin dimers. PM050489, possibly acting like a hinge at the association interface between tubulin heterodimers, reshapes Mg^2+^-induced 42 S tubulin double rings into smaller 19 S single rings made of 7 ± 1 αβ-tubulin dimers. PM060184-resistant mutants of *Aspergillus* *nidulans* map to β-tubulin Asn100, suggesting a new binding site different from that of vinblastine at the associating β-tubulin end^[227]^. PM060184 is currently undergoing clinical trials. A Phase I study with monotherapy has been completed, another one in combination with gemcitabine is ongoing and a Phase II monotherapy study is active but not recruiting.

SSE15206 is a synthetic chalcone derivative that binds to the colchicine binding site and induces G2/M mitotic arrest in a time- and dose-dependent manner in various cell systems due to failure of microtubules to polymerize, finally leading to apoptosis via induction of p53^[228]^. SSE15206 is not a substrate of P-gp efflux pumps and therefore is able to overcome multi-drug resistance. The drug is still under preclinical investigation.

## Conclusion

In conclusion, one could speculate what the best steps in avoiding or overcoming cancer resistance might be. An appropriate starting point is the great heterogeneity of tumors within the same class. As a consequence, the “one fits all” approach for cancer treatment should then be considered obsolete. The future needs to and will move to individualized therapies that have to be established following a thorough analysis of the type and status of the tumor and after having evaluated a host of biomarkers. In an ideal case, the treatment of an individual patient will be individually tailored according to the “molecular signature” of the biomarkers covering protein, RNA and DNA markers. Combinations of synergistic drugs or compounds with orthogonal mechanisms of action and sequences of complementary treatments - kill, sensitize, kill - will have to be considered. The problem of combinations is that cell-line models are not very indicative of activity/resistance if the cells have been passaged for some time. Combinations therefore still might have to be tried out in clinical studies, which certainly is limited by time and costs, in particular if more than two drugs are combined. Special care should also be taken in any future treatment considerations to cover any dormant (cancer stem) cells in the treatment schedule. Resistance can only be overcome if treatment uses the same tricks as the cancer cells do, switch to another pathway if the applied path is blocked, which, of course is very cumbersome, since the number of possible pathways is very large. However, since the development of resistance mechanisms will in future be predictable from genomic and proteomic profiles, and since sophisticated methods to measure and then tackle these mechanisms in patients will increasingly be available, the chances to finally overcome resistance are real.

## Declarations

### Copyright

© The Author(s) 2019.
